# Supplementary material for: RNA-Seq Profiling of Neutrophil-Derived Microvesicles in Alzheimer’s Disease Patients Identifies a miRNA Signature That May Impact Blood–Brain Barrier Integrity
Source: Int J Mol Sci. 2022 May 25;23(11):5913. doi: 10.3390/ijms23115913 (PMC9180128; doi:10.3390/ijms23115913)
Supplement: Supplementary file 1 [file ijms-23-05913-s001.zip › ijms-1725082-supplementary.pdf]

Supplementary Table S1. Total significantly dysregulated miRNA in NMV from AD patients.

Up-regulated miRNA	log2FC	*p*-value	Down-regulated miRNA	log2FC	*p*-value
hsa-miR-652-5p	2.82	0.00000132	hsa-miR-4485-3p	-1.19	0.00000024
hsa-let-7a-3p	2.34	0.00000358	hsa-miR-136-3p	-1.49	0.000144
hsa-miR-4443	2	0.0000198	hsa-miR-584-5p	-1.02	0.000288
hsa-miR-758-5p	3.52	0.0000399	hsa-miR-6886-3p	-2.81	0.000402
hsa-miR-2277-5p	2.8	0.000188	hsa-mir-7704	-0.73	0.000547
hsa-miR-20b-5p	1.92	0.000255	hsa-mir-548ac	-2.222	0.00105
hsa-miR-548at-5p	2.83	0.000542	hsa-miR-622	-2.75	0.00182
hsa-miR-6825-3p	2.25	0.00057	hsa-miR-126-5p	-0.79	0.00195
hsa-miR-664a-3p	2.05	0.000623	hsa-miR-136-5p	-1.2	0.0035
hsa-miR-4516	1.06	0.00093	hsa-miR-3912-3p	-2.43	0.00419
hsa-miR-4649-3p	2.67	0.00129	hsa-miR-330-3p	-1.32	0.0075
hsa-miR-4732-3p	2.25	0.00148	hsa-miR-28-3p	-0.73	0.0088
hsa-mir-4449	1.567	0.0015	hsa-miR-4644	-2.04	0.00903
hsa-miR-6750-3p	2.55	0.00182	hsa-miR-7845-5p	-2.43	0.0098
hsa-miR-18a-3p	1.31	0.00218	hsa-miR-151a-3p	-1.47	0.01
hsa-let-7g-5p	1.29	0.00223	hsa-miR-10399-5p	-1.03	0.01
hsa-miR-3651	1.31	0.00225	hsa-miR-26a-5p	-0.75	0.01
hsa-miR-1303	1.02	0.00227	hsa-miR-30e-5p	-1.01	0.01
hsa-miR-5701	1.39	0.00231	hsa-miR-320c	-0.94	0.01
hsa-miR-502-3p	1.59	0.0024	hsa-miR-342-3p	-0.8	0.01
hsa-miR-16-2-3p	1.06	0.00263	hsa-miR-21-5p	-0.69	0.0106
hsa-miR-345-5p	1.23	0.00331	hsa-mir-655	-1.782	0.0128
hsa-miR-664b-3p	1.09	0.00358	hsa-mir-495	-2.074	0.0131
hsa-let-7g-3p	2.59	0.00369	hsa-miR-128-3p	-0.64	0.0134
hsa-miR-16-1-3p	3.52	0.00369	hsa-miR-1277-3p	-2.29	0.0157
hsa-miR-6503-5p	2.52	0.00383	hsa-miR-130a-3p	-0.75	0.02
hsa-miR-454-3p	1.13	0.00453	hsa-miR-148b-3p	-0.82	0.02
hsa-miR-1246	0.84	0.00485	hsa-miR-4668-3p	-1.99	0.0212
hsa-miR-324-3p	1.26	0.00522	hsa-miR-1193	-2.17	0.0245
hsa-miR-576-5p	0.96	0.00557	hsa-mir-671	-1.462	0.028
hsa-miR-1-3p	2.16	0.00592	hsa-miR-32-5p	-1.31	0.03
hsa-miR-424-3p	1.02	0.00604	hsa-miR-548av-5p	-0.96	0.03
hsa-miR-3195	0.79	0.00625	hsa-miR-548k	-0.96	0.03
hsa-let-7e-5p	1.8	0.00677	hsa-miR-665	-1.88	0.03
hsa-mir-30c-3	1.448	0.00692	hsa-miR-7113-5p	-1.92	0.0351
hsa-miR-548ag	2.35	0.00796	hsa-miR-539-3p	-1.23	0.04
hsa-miR-181d-5p	1.14	0.00932	hsa-miR-12136	-0.8	0.04
hsa-miR-1258	2.2	0.0102	hsa-miR-30d-5p	-0.81	0.04
hsa-mir-106a	0.99	0.0109	hsa-miR-376b-3p	-1.35	0.0405
hsa-mir-181b-1	1.148	0.0109	hsa-miR-130b-3p	-1.2	0.041
hsa-miR-374c-5p	1.78	0.0112	hsa-miR-3181	-1.57	0.0495
hsa-miR-183-5p	1.25	0.012			
hsa-miR-7-1-3p	1.21	0.0127			

hsa-miR-4698	1.81	0.0141			
hsa-miR-6852-5p	1.93	0.0141			
hsa-miR-194-3p	2.25	0.0156			
hsa-miR-144-5p	0.99	0.0158			
hsa-miR-5695	1.9	0.0167			
hsa-miR-27a-5p	1.12	0.0171			
hsa-miR-30c-5p	0.88	0.0177			
hsa-miR-6777-5p	1.11	0.0179			
hsa-miR-93-5p	0.87	0.0194			
hsa-miR-424-5p	1.05	0.0195			
hsa-miR-500a-3p	0.68	0.02			
hsa-miR-7705	2.07	0.0201			
hsa-miR-4770	1.95	0.0208			
hsa-miR-363-3p	0.87	0.0209			
hsa-let-7f-1	1.1459	0.0215			
hsa-miR-501-5p	1.27	0.0217			
hsa-mir-618	1.289	0.0218			
hsa-miR-339-3p	0.99	0.0220			
hsa-mir-641	1.57	0.0242			
hsa-miR-215-5p	2.14	0.0245			
hsa-miR-361-3p	1.17	0.0248			
hsa-miR-3146	0.88	0.0253			
hsa-miR-6879-5p	2.07	0.0259			
hsa-miR-2054	1.95	0.0261			
hsa-miR-6501-3p	1.5	0.0268			
hsa-miR-191-3p	1.26	0.0273			
hsa-mir-371b	1.104	0.0274			
hsa-mir-181a-2	1.0366	0.0275			
hsa-miR-23b-3p	1.13	0.0278			
hsa-miR-140-3p	0.73	0.0281			
hsa-miR-6832-3p	1.7	0.0284			
hsa-miR-3168	0.83	0.0285			
hsa-miR-103a-2-5p	1.15	0.0291			
hsa-miR-181c-5p	0.53	0.03			
hsa-miR-3141	1.25	0.03			
hsa-mir-194-2	2.26	0.0301			
hsa-mir-3916	1.524	0.0306			
hsa-mir-548aw	1.494	0.0333			
hsa-miR-656-5p	1.93	0.0337			
hsa-miR-425-5p	0.83	0.0341			
hsa-mir-5010	1.985	0.0347			
hsa-miR-875-5p	1.25	0.0351			
hsa-mir-99a	0.989	0.0351			
hsa-miR-224-3p	1.68	0.0355			
hsa-miR-4298	1.69	0.0358			
hsa-miR-4488	0.82	0.0358			
hsa-miR-20a-5p	0.69	0.0359			

hsa-miR-616-5p	1.53	0.0359			
hsa-mir-6084	1.338	0.0361			
hsa-mir-6877	1.438	0.0361			
hsa-miR-223-3p	0.52	0.0372			
hsa-miR-548f-5p	1.58	0.0378			
hsa-miR-34b-5p	1.61	0.0385			
hsa-mir-3613	1.041	0.0386			
hsa-miR-6819-3p	1.53	0.0386			
hsa-miR-766-3p	0.96	0.0398			
hsa-mir-24-1	0.977	0.0413			
hsa-miR-182-5p	1.3	0.0416			
hsa-miR-4802-3p	1.95	0.0424			
hsa-miR-221-5p	1.08	0.0425			
hsa-mir19b-1	0.92	0.0431			
hsa-miR-210-3p	1.12	0.0438			
hsa-let-7d	1.003	0.044			
hsa-miR-877-5p	1.52	0.0441			
hsa-miR-19b-3p	0.51	0.0457			
hsa-miR-3200-5p	1.9	0.0463			
hsa-miR-4286	0.82	0.0472			
hsa-mir-374b	1.025	0.0473			
hsa-mir181a-1	0.909	0.0503			
hsa-mir-708	1.019	0.0504			
hsa-miR-199b-5p	0.84	0.0507			
hsa-mir-625	1.201	0.0521			
hsa-mir-5047	0.999	0.0525			
hsa-mir-101-2	0.71	0.0543			
